# White-to-Beige and Back: Adipocyte Conversion and Transcriptional Reprogramming

**DOI:** 10.3390/ph17060790

**Published:** 2024-06-16

**Authors:** Stanislav Boychenko, Vera S. Egorova, Andrew Brovin, Alexander D. Egorov

**Affiliations:** 1 Gene Therapy Department, Center for Translational Medicine, Sirius University of Science and Technology, 354340 Sirius, Russia; bojchenko.ss@talantiuspeh.ru (S.B.); brovin.an@talantiuspeh.ru (A.B.); 2 Biotechnology Department, Center for Translational Medicine, Sirius University of Science and Technology, 354340 Sirius, Russia

**Keywords:** adipose tissue, adipogenesis, anti-obesity, adipose browning, thermogenesis, transcription factors, PPAR gamma, phytochemicals, aryl hydrocarbon receptor, endocrine disruptors

## Abstract

Obesity has become a pandemic, as currently more than half a billion people worldwide are obese. The etiology of obesity is multifactorial, and combines a contribution of hereditary and behavioral factors, such as nutritional inadequacy, along with the influences of environment and reduced physical activity. Two types of adipose tissue widely known are white and brown. While white adipose tissue functions predominantly as a key energy storage, brown adipose tissue has a greater mass of mitochondria and expresses the uncoupling protein 1 (*UCP1*) gene, which allows thermogenesis and rapid catabolism. Even though white and brown adipocytes are of different origin, activation of the brown adipocyte differentiation program in white adipose tissue cells forces them to transdifferentiate into “beige” adipocytes, characterized by thermogenesis and intensive lipolysis. Nowadays, researchers in the field of small molecule medicinal chemistry and gene therapy are making efforts to develop new drugs that effectively overcome insulin resistance and counteract obesity. Here, we discuss various aspects of white-to-beige conversion, adipose tissue catabolic re-activation, and non-shivering thermogenesis.

## 1. Introduction

Obesity is excessive fat deposition, the primary cause of which is metabolic imbalance. According to the World Health Organization reports [1] and latest studies, the number of individuals with obesity has doubled during the last 30 years; therefore, obesity is becoming prevalent across continents and diverse ethnic groups [2]. Currently, one in eight people is obese. Obesity is a major risk factor for type 2 diabetes, which remains among the leading causes of death and disability [3,4]. During the COVID-19 pandemic, the problem of the “double burden of disease” has come to the fore: severe acute respiratory infection emerged as a leading cause of death among patients with obesity and type 2 diabetes [5,6].

From its establishment at the embryonic stage, adipose develops throughout the entire lifetime of an individual [7]. Abnormal expansion of adipose tissue relative mass results in pathologies such as obesity and cardiovascular diseases. The etiology of obesity is multifactorial and combines hereditary factors and the influence of social factors, such as malnutrition, along with reduced physical activity [8].

Adipose tissues represent a specific kind of connective tissue, considered to be a main depot for lipid accumulation, and also play a major role in the regulation of systemic metabolism [9]. Indeed, adipose sequesters the content of low-density lipoproteins, accumulates triglycerides in its lipid droplets, absorbs glucose from the bloodstream, and is able to transform it into lipids [10]. In addition to its primary function of major energy storage, adipose tissue serves as an endocrine organ; it secretes various molecules that affect systemic responses and metabolism [11].

Earlier, two canonical types of adipose tissue were described: white and brown adipose [12,13]. Unlike cells of white adipose tissue, brown adipocytes have a greater mass of mitochondria and express gene coding for uncoupling protein, which allows thermogenesis and rapid catabolism [14]. Even though white and brown adipocytes are of different origins, activation of the brown adipocyte differentiation program (e.g., as a consequence of constant sympathetic stimulation) in white adipose tissue leads to the appearance of clusters of “beige” adipocytes [15].

During chronic cold exposure, cells of white adipose tissue undergo “beigeing” and convert into beige adipocytes, characterized by thermogenesis and intensive lipolysis. Vice versa, during a prolonged positive energy balance, beige adipose can lose its thermogenic capability and undergo transdifferentiation into white adipose tissue [16,17].

A considerable time has elapsed since it was proposed that beige adipose tissue has therapeutic relevance [18]. Acknowledging the potential of molecular-based therapies in the treatment of obesity and associated diseases, we summarize the current knowledge about white-to-beige adipose conversion, discuss major transcription factors involved in the process, and focus on small molecules that participate in transdifferentiation, along with other possibilities to stimulate this conversion.

## 2. Types of Adipose

Adipose, comprising 20 to 25% of the total body weight in healthy individuals, reaches a considerable proportion of the body mass. Adipose tissue is composed of numerous cell types, including mature adipocytes, progenitor cells, stromal cells, and endothelial cells. In addition, adipose tissue contains a wide variety of immune cells that play a pivotal role in the homeostasis and normal functioning of adipose tissue [19]. Despite the multiplicity of cell types, the main cellular component of adipose is adipocytes.

In the early years of study, human adipose tissue was categorized into two functionally diverse types: white and brown adipose [20]. White adipose tissue is the most abundant type of adipose in adults, and the occurrence of obesity is associated with white adipose expansion [21]. White adipose tissue (WAT) resides in specific depots in the body, subcutaneous and visceral, classified based on their localization [22]. By contrast, brown adipose tissue (BAT) is predominantly present in infants and has a lesser amount in adults, concentrating mostly in the armpits, and the interscapular and supraclavicular regions [23]. Human brown adipose tissue ensures the survival of neonates during the first weeks after birth. In contrast to WAT, brown adipose tissue has a characteristic dark coloration due to the higher concentration of mitochondria. Mature brown adipocytes are characterized by thermogenin expression (uncoupling protein 1, UCP1). UCP1 is capable of transporting protons to the matrix without ATP synthesis, which leads to the release of energy in the form of heat. Since, together with the proton, UCP1 transfers free fatty acids used for oxidation in mitochondria, an increase in the concentration of free fatty acids leads to a higher conductance of protons [24]. Thus, BAT has an increased rate of catabolism, which results in non-shivering thermogenesis, and the body accesses the energy contained in deposited lipids.

Despite having many similarities in appearance and secretory activity, white and brown adipose tissues differ radically on the cellular level. In particular, brown adipocytes, having multiple lipid droplets (multilocular), are morphologically distinctive from unilocular mature white adipocytes [25]. Moreover, it is widely accepted that brown and white adipocytes originate from cells of different lineages [26]. While white adipocytes arise from multipotent progenitor cells expressing the surface marker platelet-derived growth factor receptor α (PDGFRα) [27], brown adipocytes originate from progenitors that characteristically express myogenic factor 5 (*Myf5+*), also specific to skeletal muscle cells [26]. However, there is evidence that PDGFRα+ cells participate in the development of both WAT and BAT [28], as well as that Myf5+ precursors can give rise to a broader group of cells than it was thought [29], yet the majority of brown and white adipocytes surely have distinct origins.

Brown adipose tissue was thought to be absent in adults [30], but with the efforts of nuclear medicine, thermogenic adipocytes have been found to be active in some adults [31,32,33,34]. According to the latest research, adult BAT is normally activated by cold, has a high prevalence without sexual dimorphism, and is negatively related to body fat percentage; however, it declines with age and BMI [35]. Returning to its protective role, adults with metabolically healthy overweight or obesity present higher BAT volumes and are more thermogenic compared with unhealthy obese individuals [36]. Therewith, brown adipose contributes to the enhanced consumption of glucose [37]. Together with epidemiological studies showing that the presence of brown adipose tissue correlates with lower levels of type 2 diabetes, dyslipidemia, coronary heart disease, and hypertension [38], these findings allow us to embrace the hope of a prospective drug being invented.

For all that, white and brown are not the only types of adipocytes. As was mentioned above, clusters of another kind of adipocyte tend to appear in white adipose tissue. Cold acclimation increases non-shivering thermogenesis in brown adipose and induces the appearance of brown-like loci in white adipose tissue [39,40]. Thermogenic cells isolated from the murine white fat depots occur as something in between, neither white nor brown, but “beige” cells, also called brite (brown-in-white) [41]. Similarly to white adipocytes, beige cells exhibit low basal expression of *UCP1*, but upon stimulation by cold or other stimuli, they respond by increasing the respiratory rate and *UCP1* expression [42]. Beige adipocytes originate predominantly from the Myf5-negative lineage, in contrast to brown adipocytes [43]. It was also revealed that some beige adipocytes have myosin heavy chain 11 (Myh11), which is a marker of smooth muscle cells [44]. The emergence of cold-induced thermogenic adipocytes is determined predominantly by white-to-beige adipocyte transdifferentiation. Thus, the origin of beige adipocytes is close to white, but their fate is pre-determined by the molecular factors that are able to induce this white-to-beige conversion.

There is a complex interplay in adipose tissue between adipocytes, their progenitors, and, last but not least, resident immune cells [45]. Adipocytes secrete multiple factors mediating interactions important for immune cells residing in adipose, stimulating their proliferation and possible interchange [46]. There is clear evidence that immune cells, such as adipose tissue macrophages and lymphoid cells, can influence metabolic homeostasis through adipose tissue. For instance, adipose tissue macrophages originate from yolk sac progenitors, self-renew, and become permanently resident for the rest of life [47]. Macrophages are pivotal in obesity-associated inflammation and metabolic diseases. Adipose tissue macrophages secrete small extracellular vesicles able to improve glucose tolerance and increase insulin sensitivity in adipocytes, myotubes, and primary mouse and human hepatocytes [48].

Moreover, it was revealed that another group of cells arising during fetal development from the yolk sac, innate lymphoid cells (ILC2s) [49], could promote the beigeing process in WAT [50]. It is likely that decreased ILC2 responses in human WAT is a conserved characteristic of obesity. Interleukin IL-33, critical for the maintenance of ILC2s in WAT, was associated with the recruitment of uncoupling protein 1 (UCP1)+ beige adipocytes in WAT. IL-33-induced beigeing was connected to the production by ILC2s of methionine-enkephalin peptides, which act directly to upregulate Ucp1 gene expression in adipocytes and promote beigeing in vivo [50].

Other circulating factors, such as myokines FGF21 [51], BMP7 [52,53], and exercise-induced irisin [54], are known to trigger the beigeing of WAT as well. The fact is that these molecules are also normally produced by adipose tissue and act in an autocrine manner [55,56,57]. There is also experimental proof that ILC2s residing in WAT produce BMP7 and are able to induce the white-to-beige transdifferentiation of adipose [58].

The present section highlights aspects related to the cellular mechanisms substantial for the normal functioning of adipose tissue, in this way determining molecular factors that can trigger white-to-beige transdifferentiation of adipose.

## 3. Transcriptional Events behind Adipogenesis

Adipocyte differentiation, also known as adipogenesis, is the process by which fibroblast-like precursor cells (preadipocytes) transform into mature differentiated adipocytes under the influence of adipogenic stimuli such as insulin and glucocorticoid agonists. This process may be significantly affected by an individual’s genetic background and by specific small molecules, such as pharmaceuticals, phytochemicals, and pollutants.

Excessive fat accumulation is connected with both hypertrophy (an increase in adipocyte size) and hyperplasia (an increase in adipocyte numbers) and develops as a result of positive energy balance and the rate of adipocyte differentiation. The state of equilibrium between differentiated adipocytes and stromal precursor cells in adipose tissue is regulated by the zinc finger cell fate regulator Zfp521 [59]. Zinc finger protein Zfp423 influences both the early determination of preadipocytes and the terminal differentiation of adipocytes [60,61,62]. During differentiation of preadipocytes, Zfp423 controls the expression of key adipogenic regulators peroxisome proliferator-activated receptor γ (PPARγ) and CCAAT-enhancer-binding protein α (C/EBPα) [62,63]. However, in mature white adipocytes, Zfp423 maintains an energy-storing phenotype by suppressing the thermogenic gene program [64].

In response to adipogenic stimuli, transcription factors that regulate the expression of numerous genes involved in adipocyte differentiation are activated. The transcriptional cascade that controls differentiation has been studied extensively [12,65]. Transcription factors determine cell fate by regulating the expression of multiple genes, including genes coding for other transcription factors that determine subsequent specialization.

It was shown that the process of adipogenesis is tightly regulated by the coordinated actions of transcription factors and epigenetic regulators that determine cell fate. The peroxisome proliferator-activated receptor γ and CCAAT-enhancer-binding protein α are the main adipogenic transcription factors [66].

In recent years, numerous transcriptional and epigenetic regulators of adipogenesis have been identified in multiple studies [67]. Based on the composition of transcription complexes involved in the process and the genes they control, the adipogenic cascade is divided into two main sequential “waves” that drive the adipogenic program [68]. The “first wave” is triggered by adipogenic stimuli and includes factors such as C/EBPβ and C/EBPδ, Krüppel-like factors (KLFs), and CREB (cAMP-responsive element-binding protein). The transcription factors of the “first wave” induce the expression of the “second wave” factors, *C/EBPα* and *PPARγ*, which in turn cooperatively bind the regulatory sequences and control the expression of adipocyte-specific genes [69].

CCAAT/enhancer-binding protein β (C/EBPβ) plays an important role in the initiation and regulation of adipogenesis, acting as an early transcription factor that triggers the cascade of gene expression that is necessary for adipocyte differentiation. C/EBPβ activation is regulated by several signaling pathways, including the mitogen-activated protein kinase (MAPK) and the glycogen synthase kinase 3β (GSK3β) pathways [70]. Glucocorticoids are another important regulator of C/EBPβ. They enhance the acetylation of C/EBPβ, which increases its transcriptional activity. This effect is mediated through the interaction of C/EBPβ with acetyltransferases like p300/CBP-associated factor (PCAF) [71]. Upon activation, C/EBPβ upregulates the expression of genes of the “second wave” like a peroxisome proliferator-activated receptor gamma (PPARγ), C/EBPα, and others [72].

Another member of the C/EBP family of transcription factors, C/EBPδ, plays a significant role in adipocyte differentiation. Together with *C/EBPβ*, induction of *C/EBPδ* expression occurs at the early stages of adipocyte differentiation. Moreover, C/EBPβ and C/EBPδ have a synergistic role in adipocyte differentiation both in vitro and in vivo. Primary embryonic fibroblasts from both C/EBPβ (−/−) and C/EBPδ (−/−) mice were not able to differentiate into mature adipocytes and lacked the capacity to express *PPARγ* and *C/EBPα* [73].

Our previous study revealed another transcription factor that negatively controls the adipogenic differentiation. The knockdown of Prep1 gene expression was shown to affect the conversion of murine preadipocytes from the 3T3-L1 cell line into adipocytes [74]. Decreased expression of Prep1 resulted in enhanced adipogenic differentiation and a significant increase in the insulin-sensitive glucose carrier Glut4 gene expression [75]. Prep1 downregulation resulted in a significant improvement in the ex vivo adipogenic differentiation of both adipose-derived mesenchymal stromal cells (MSCs) [76] and bone marrow-derived MSCs [77]. Presumably, the absence of Prep1 increased the binding of C/EBPβ to chromatin, as it did not affect the C/EBPβ level nor phosphorylation in 3T3-L1 cells [77]. Single cell transcriptomics revealed the increase in cells with the Brown Fat Cell Differentiation pattern of gene expression in hypomorphic Prep1^i/i^ mice suggesting that a lower dosage of Prep1 results in adipose “browning” in vivo [78].

**Figure 1:**
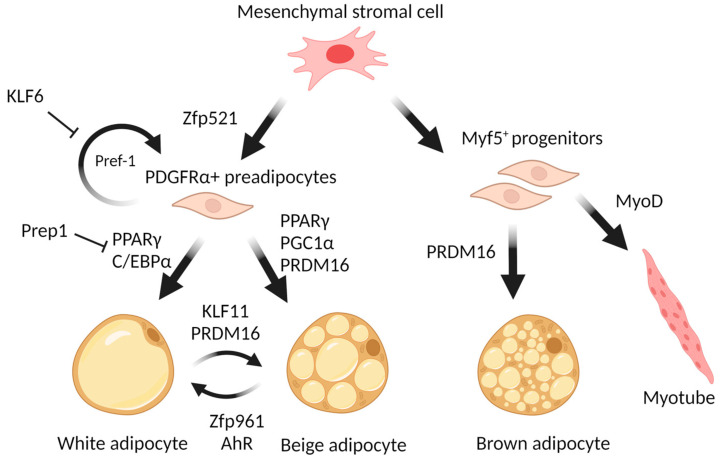
The scheme of adipocyte differentiation.

The “first wave” of the adipogenic cascade also includes Krüppel-like factors that are involved not only in adipogenesis but also in the processes of cellular growth and apoptosis. KLFs are characterized by their ability to bind to CACCC-box and GC-rich regions of DNA, through which they regulate the transcription of target genes. Several KLFs are known for their ability to promote adipogenesis. For example, KLF4 is induced by cAMP and promotes adipogenesis by controlling *C/EBPβ* expression [79]. KLF5 facilitates adipogenesis through the activation of main adipogenic transcription factors such as PPARγ and C/EBPα [80]. KLF6 promotes preadipocyte differentiation (Figure 1) by repressing a gene coding for preadipocyte factor named Delta-like 1 (Dlk1, or Pref1), which is a well-known autocrine suppressor of adipogenesis [81]. On the other hand, several KLF factors suppress adipogenesis: KLF2 inhibits the *Pparg* gene expression [82], KLF3 represses the transcription of *C/EBPα* by recruiting C-terminal binding protein (CtBP) corepressors [83], and KLF7 inhibits adipogenesis by decreasing the expression of both *PPARγ* and *C/EBPα* [84]. Importantly, there is a member of the KLF family, KLF11, that acts specifically to promote white-to-beige transdifferentiation by altering the “superenhancers” bound by PPARγ towards the set selective for beige adipocytes [85].

There were ~12,000 transcription factor hotspots identified in the early phase of adipogenesis, characterized by simultaneous and sequential binding of transcription factors [86]. These hotspots are highly enriched in superenhancer regions (expanding to several kilobases) that drive the adipogenic reprogramming of gene expression. Specifically, it was revealed that the binding of transcription factors to these superenhancers defines the process of white-to-beige transdifferentiation [85]. Apparently, the role of superenhancers in adipose beigeing highlights the importance of the mediator complex. Superenhancers are characterized by the immense binding of Mediator subunit 1 (MED1). Although MED1 is not required for the survival of embryos or for the proliferation of embryonic stem cells, it fulfills essential roles during the terminal differentiation of adipocytes. Ablation of MED1 leads to defects in the development of both brown and white adipocytes and lipodystrophy [87].

Cyclin C (CCNC) is one of the subunits of the Mediator complex, which has demonstrated its importance for adipogenic regulation. CCNC deficiency impaired the proliferation of brown fat progenitors during embryogenesis. But its ablation did not affect either brown adipogenesis or cell death. Moreover, the deficiency of CCNC was shown to reduce the accumulation of lipids in differentiated brown adipocytes age-dependently. CCNC in adipocytes is required for lipogenic gene expression through the activation of the C/EBPα/GLUT4/ChREBP axis [88].

CREB-TF (CREB, cAMP response element-binding protein) is a transcription factor that binds to cAMP response elements, specific DNA sequences found upstream of genes induced after cAMP elevation. Activation of CREB is predominantly mediated by the cAMP signaling pathway. Once activated, CREB promotes adipogenesis by enhancing the transcription of *C/EBPβ* by binding to its promoter [89]. Moreover, CREB activation is both necessary and sufficient to initiate adipogenesis in the 3T3-L1 model cell line of preadipocytes. *CREB* expression is stimulated by several adipogenesis-inducing agents, such as insulin, dexamethasone, and dibutyryl cAMP. In addition to C/EBPβ, CREB directly binds to the promoters of adipocyte-specific genes such as peroxisome proliferator-activated receptor gamma 2 (*PPARγ2*, the shorter isoform of *PPARγ*) and fatty acid-binding protein 4 (*FABP4*) [90]. Depletion of CREB and its closely related ATF-1 (Activating Transcription Factor 1, cyclic AMP-dependent transcription factor) expression in 3T3-L1 preadipocytes leads to their inability to differentiate into mature adipocytes [91].

The “second wave” of differentiation includes major factors such as PPARγ and C/EBPα. It is widely accepted that the main role in the execution of an adipogenic program belongs to the nuclear receptor PPARγ. Its expression is a necessary and sufficient requirement for differentiation towards the mature adipocyte state [92]. Ectopic expression of *PPARγ* is sufficient to induce adipocyte differentiation in fibroblasts, and so far, no transcription factor has been identified that is able to promote adipogenesis in the absence of PPARγ [93,94]. Furthermore, PPARγ not only triggers adipocyte differentiation but also modulates the metabolic functions of mature adipocytes. It plays a crucial role in lipid uptake and storage, and its activity is modulated by various post-translational modifications, which affect its stability and interaction with coactivators and corepressors. Upon activation, PPARγ forms heterodimers with the retinoid X receptor (RXR) and binds to specific DNA sequences known as peroxisome proliferator response elements (PPREs) within the regulatory regions of target genes. It upregulates the expression of key transcription factors such as *C/EBPα* (CCAAT/enhancer-binding protein alpha) and *C/EBPβ*, which initiate the differentiation cascade. In addition, PPARγ directly stimulates the expression of adipocyte-specific genes, including those of adiponectin, leptin, and fatty acid-binding protein 4 (*FABP4*) [95]. These genes are essential for lipid transport and accumulation, regulation of homeostasis, systemic insulin sensitivity, and adipocyte function. More than 60% of the genes upregulated during the process of adipogenesis have binding sites for both *PPARγ* and *C/EBPα* within 50 kb of the transcription start site [96]. It was defined that the total number of PPARγ-binding sites exceeds 52,000, of which 4439 were attributed either to white or beige adipocytes (2228 white-selective and 2211 beige-selective) [85].

Although PPARγ has an effect on the expression of a large number of genes, much attention has been paid to the study of the interaction between PPARγ and the transcriptional regulator PR domain-containing protein 16 (PRDM16). PPARγ directly recruits PRDM16 to form a transcriptionally active complex that triggers a browning program in WAT. PPARγ also recruits other factors such as Histone-lysine N-methyltransferase (EHMT1) and Early B-cell factor (EBF2) that can be present in the PPARγ/PRDM16 complex and enhance its function [97]. Transcriptional complexes formed by PPARγ with PRDM16, EBF2, and EHMT1 determine the beigeing of white adipose tissue, and maintain the unique functions of adaptive thermogenesis and energy homeostasis controlled by the PPARγ/PRDM16/PGC1α complexes in the brown/beige cells.

PRDM16 plays a pivotal role in the determination and differentiation of brown and beige adipocytes. During transdifferentiation of myoblasts into brown adipocytes, PRDM16 serves as a molecular switch, and its PR domain determines alterations in CpG methylation of myogenic factors [98]. PRDM16 enhances transdifferentiation into BAT by interacting with PPARγ and activating its transcriptional function. Loss of *PRDM16* expression in BAT precursors promotes muscle differentiation [26]. *PRDM16* expression in subcutaneous white adipocytes is a determinant of a brown fat-like gene program and thermogenesis in these tissues [99]. The *Prdm16* gene is required in young mice to suppress the expression of white adipocyte-selective genes in BAT through recruitment of the histone methyltransferase Ehmt1 (G9a-like protein), the enzyme that provides repressive modifications of chromatin [100]. Loss of *EHMT* leads to a brown fat deficiency and induces muscle differentiation in vivo through demethylation of histone 3 lysine 9 of the muscle-selective gene promoters [97]. In order to ensure the induction of browning, PRDM16 should form a transcriptional complex with C/EBPβ. This complex enhances the function of C/EBPβ as a transcriptional activator, inducing the expression of other transcription factors and coactivators, such as *PPARγ* and *PGC1α* [101]. As well, PRDM16 physically binds to MED1 (Mediator Complex Subunit 1) and recruits it to PPARγ superenhancers at beige-selective genes [102]. Other proteins can regulate PRDM16 stability. For example, in [103], it was demonstrated that the CUL2–APPBP2 ubiquitin E3 ligase complex catalyzes the polyubiquitination and degradation of PRDM16 protein. Inhibition of this complex extends the PRDM16 protein half-life and stimulates adipocyte browning. On the other hand, CBX4 (Chromobox 4), a polycomb group protein, is a SUMO E3 ligase for Prdm16 [104]. An increased expression level of CBX4 in adipose tissue during cold exposure leads to sumoylation of Prdm16 at the lysine 917 residue and thus blocks its ubiquitination-mediated degradation. Regulating the interaction between PRDM16 and PPARγ is one of the ways for cells to enhance or suppress a WAT browning. Qi-Xiang Ma and colleagues showed [105] that knockout of *Bcat2* (Branched Chain Aminotransferase 2) leads to an increase in inguinal WAT browning and thermogenesis through the suppression of acetylation of PRDM16 at K915. This modification disrupts the interaction between PRDM16 and PPARγ. PexRAP is a peroxisomal lipid synthetic enzyme interacting both with PPARγ and PRDM16 that can disrupt the PRDM16-PPARγ complex [106]. Transcription factor Hlx (H2.0-Like Homeobox) through Prdm16-mediated co-activation drives WAT browning [107]. It was described that Prdm16 interacts with the transcription factor Hlx, which increases the expression of genes specific to thermogenic adipocytes by stabilizing Prdm16 in response to β3-adrenergic signaling. Hlx is involved in the regulation of white adipose tissue beigeing, and may be seen as a possible molecular target.

Peroxisome proliferator-activated receptor gamma coactivator 1-alpha (PGC-1α) is a key regulator of energy metabolism in a cell. PGC-1α is activated by the action of the cAMP signaling pathway (PKA-p38/MAPK) and physically interacts with PPARγ, PPARα, and other nuclear factors. PGC-1α influences genes related to energy metabolism, including mitochondrial biogenesis, oxidative phosphorylation, and gluconeogenesis, and it is expressed mostly in tissues that require an elevated amount of energy. Adipose-specific overexpression of PGC-1α promotes the browning of white adipose tissue and enhances mitochondrial biogenesis and respiration [108].

EBF2 is a member of the early B-cell factor family of transcription factors and is known to play critical roles in B-cell development, neuronal development, and immune cell function. Several studies have highlighted the importance of EBF2 in adipocyte differentiation [109,110]. *EBF2* is highly expressed in brown adipose. It has been demonstrated that EBF2 promotes the expression of genes associated with mitochondrial biogenesis and thermogenesis (*UCP1*). Suzanne Shapira and her colleagues demonstrated that EBF2 functions as a transcriptional activator of the histone acetylation and methylation reader DPF3 (double PHD fingers 3), which recruits the BAF chromatin remodeling complex to brown adipocyte-specific gene promoters [111]. This mechanism leads to the induction of brown adipocyte genes’ expression and the acquisition of a brown-like phenotype in white adipocytes. Additionally, EBF2 binds to PPARγ target genes, reprogramming cells to brown adipocytes when expressed in myoblasts [110]. Both EBF1 and EBF2 cooperate with estrogen-related receptor α and PGC-1 α to promote Ucp1 transcription [112].

It is known that members of the forkhead box factors (Fox) family are largely involved in adipose tissue functioning. In particular, the main intracellular target of insulin signaling, FoxO1, inhibits the expression of *UCP1* [113]. In a quite recent study, it was demonstrated that hepatic FoxO1 suppresses systemic Fgf21 secretion, promotes the whitening of brown/beige adipose tissue, and impairs glucose metabolism [114]. Most recently, an in vivo study utilizing post-developmental adipose-specific conditional deletion of FoxO1 in mice showed an increase in lineage plasticity and adipose browning [115]. Another member of the forkhead box family, FoxP1, inhibits brown/beige adipocyte differentiation and thermogenesis by repressing the transcription of the β3-adrenergic receptor (β3-AR) [116].

Conversely, FoxA3 contributes positively to adipocyte differentiation, modulating *PPARγ* expression both in vitro and in vivo [117]. Lately, it has been shown that FoxP4 is expressed in adipose tissue and directly regulates the *UCP1* expression level via binding to the response element upstream of the *UCP1* transcription start site [118]. The binding sites of *FoxP4* were also identified upstream of the promoter region of the *Ppargc1a* gene, which codes for PGC1α [119]. Importantly, the FoxP4 level correlates with the expression level of such BAT marker genes as PRDM16, PGC1α, and Elovl3 [119].

Cell death-inducing DNA fragmentation factor-like effector A (CIDEA) is a member of the CIDE family of proteins, which are characterized by the presence of a conserved CIDE-N domain. In adipocytes, CIDEA is a protein associated with lipid droplets that promotes lipid droplet fusion in brown adipocytes [120]. In beige adipocytes, CIDEA transcriptionally regulates UCP1 by inhibiting LXRα repression of UCP1 enhancer activity and increasing PPARγ binding to the UCP1 enhancer [121].

Understanding the role of miRNAs in transcriptional regulation of adipocyte browning could offer new opportunities for drug development.

Some miRNAs serve as positive regulators of browning. miR-455 promotes brown adipocyte differentiation and thermogenesis by activating AMPKα1 and targeting adipogenic suppressors Necdin and Runx1t1 [122]. Runx1t1 is also the target for another miRNA—miR-193b [123]. Overexpression of miR-30b/c stimulates UCP1 expression through the suppression of transcriptional corepressor receptor-interacting protein 140 (Rip140) [124] and mediating PPARγ activity [125]. Transducer of ErbB-2.1 (Tob1) repression by miR-32 results in increased serum Fibroblast Growth Factor 21 (FGF21) levels that stimulate browning of scWAT in the response to prolonged cold exposure [126]. Inhibition of miR-182 or miR-203 results in a reduction in brown adipocyte marker mRNAs, such as Ucp1, Pgc1a, Cidea, and Ppara [127]. The expression of miR-129 results in an increase in UCP1 expression through the targeting of its inhibitors—Igf2 (insulin-like growth factor 2) and Egr1 (Early growth factor response 1) [128]. MiR-669a-5p mimic significantly enhances the expression of Pgc-1α and Ucp1 in 3T3-L1 cells and promotes adipogenic differentiation of C3H10T1/2 cells [129].

Other miRNAs act as negative regulators of browning. Overexpression of miR-27 in brown preadipocytes suppresses the expression of *Prdm16, Ucp1, Creb, and Pparα* by targeting the 3′ UTR of *Prdm16* and *Pparα* [130]. miR-34a attenuates FGF21 signaling through the downregulation of FGFR1 and SIRT1 that increases PGC-1α acetylation, which results in the suppression of PGC-1α activity [131]. miR-155 suppresses the development of adipogenic and thermogenic programs by targeting Cebpβ [132]. miR-133 downregulates *PRDM16* expression, thereby suppressing the brown fat differentiation and browning [133]. Inhibition of miR-143-3p protected against the development of insulin resistance by targeting insulin-like growth factor 2 receptor (IGF2R) [134]. MiR-93 serves as a negative regulator of adipogenesis by targeting transcription factor T-box 3 (Tbx3) and Sirtuin-7 (Sirt7) [135]. MiR-378 has a dual role as an inhibitor of browning and as positive regulator of BAT [136].

Summarizing the description of transcription factors involved in adipogenesis and WAT browning, it can be concluded that increased activation and expression of genes involved in thermogenesis, the specific transcriptional pattern of brown and beige adipocytes, is an important feature of browning in WAT. This pattern includes PPARα and PPARγ, as well as their coactivators PRDM16 and PGC1-α, CIDEA, and major functional proteins such as type II iodothyronine deiodinase (DIO2) and uncoupling protein 1 (UCP1). Most of the chemicals that affect the process of transdifferentiation act upon the transcription factors involved in the white-to-beige adipose conversion.

## 4. Small Molecule Compounds and Dietary Molecules

Reprogramming of adipose cells using small molecules that act upon (activate or inhibit) molecular targets involved in BAT biogenesis is a simple, noninvasive intervention technique that is currently being actively studied [137]. This approach enables an opportunity to counteract obesity and related diseases such as type 2 diabetes mellitus, hyperlipidemia, dyslipidemia, cardiovascular disease, etc. With the use of small molecules, not only adipocytes but also fibroblasts [138], endothelial cells [139], and myoblasts [140] can be transformed into beige adipocytes.

Here, we provide a brief retrospective of the small molecules that induce adipose tissue browning via their effect on the respective transcription factors.

### 4.1. PPARs

PPARγ is able to bind numerous compounds, including its natural ligand (15-deoxy-Δ12,14 prostaglandin J2) and synthetic anti-diabetic thiazolidinediones; this binding promotes heterodimerization of PPARγ with RXR and activation of PPAR-dependent genes. Full agonism of PPARγ ligands has been shown to activate the brown adipose transcriptional program in subcutaneous white adipose tissue [141]. However, due to the specific structure of the ligand-binding site, PPARγ is able to interact both with synthetic ligands, such as rosiglitazone and pioglitazone, and many natural compounds (with lower affinity), including the flavonoids quercetin [142], kaempferol [143], and carotenoid lycopene [144,145]. The effects of the above substances include activation and upregulation of PPAR-dependent genes in various cell types: macrophages, salivary gland cells, prostate tumor cells, and adipose tissue.

**Figure 2:**
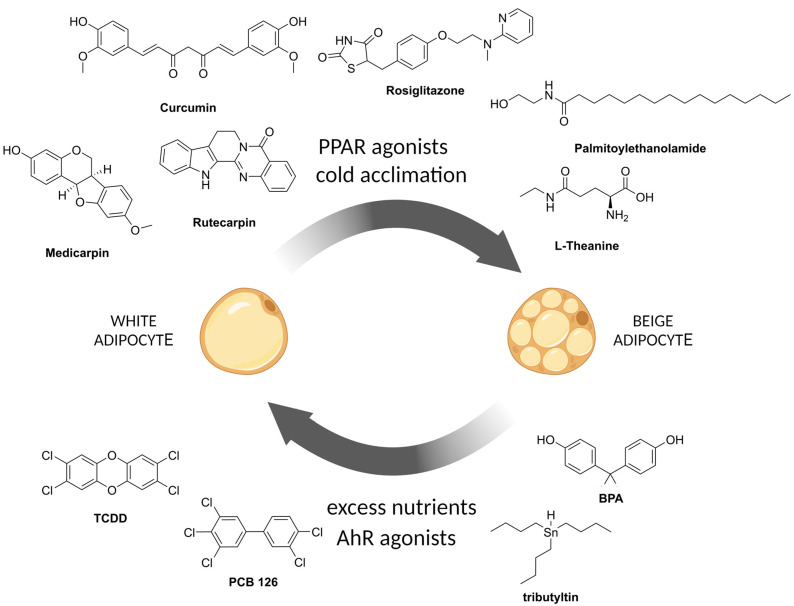
Chemical substances that affect white-to-beige adipocyte conversion.

Synthetic PPARγ activators, in particular rosiglitazone (Figure 2), increase the expression levels of brown adipocyte-specific genes (*UCP1*, *CIDEA*, *ELOVL3*, and *DIO2*) in white adipocytes via SIRT1-, PRDM16-, C/EBPα-, and PGC1α-mediated mechanisms [85]. In [85], using human white adipocytes (human multipotent adipose-derived stem (hMADS) cells) in vitro, it was demonstrated that once induced, the expression of brown adipocyte marker genes is maintained for a period up to one week independently of continued rosiglitazone administration; hence, the compound administration switches on a stable beige adipocyte gene program. However, browning induced by thiazolidinedione treatment in vivo was not associated with increased energy expenditure or weight loss [146].

Recently, dual PPARα/γ activation was described, which also promotes browning of adipose tissue in vivo and is superior to selective PPARγ activation due to a concomitant PPARα-mediated upregulation of fibroblast growth factor 21 (FGF21), which also plays an important role in adipogenesis. The most active substance of dual PPARα/γ activators, tesaglitazar, increased the *UCP1* expression level in mouse preadipocytes (up to 1700-fold) and adipocytes (up to 80-fold). Two-week long in vivo experiments proved that tesaglitazar treatment resulted in an increase in Ucp1, Pgc1a, and Cidea mRNA levels in WAT and, most importantly, in increased energy expenditure in contrast to rosiglitazone treatment. Additional benefits of tesaglitazar treatment were increased insulin sensitivity, improved dyslipidemia (2-fold reduction in both plasma triglycerides and total cholesterol levels), and improved hepatic steatosis (liver triglyceride content reduced by more than 80% in the high-dose tesaglitazar group compared to controls) [147].

It is worth noting that PRDM16 knockout significantly inhibited rosiglitazone-induced browning and completely abolished rosiglitazone-induced increases in uncoupled respiration in adipocytes. Rosiglitazone significantly increased PRDM16 protein levels in vivo both in BAT and inguinal WAT from wild type mice, and from PRDM16 transgenic mice in contrast to a non-significant or no effect on PRDM16 mRNA levels. Therefore, rosiglitazone revealed itself to be a potent stabilizer of the PRDM16 protein [141].

Sesaminol, a lignan extracted from the seeds of sesame (*Sesamum indicum*), increased expression levels of *Ucp1*, *Fabp4*, and *Pparγ*, and mitochondrial-specific genes such as Cidea, Pgc1α, Pparα, Cox8b, and Dio2 in mouse primary white adipocytes in vitro. In vivo intraperitoneal administration of sesaminol promoted the formation of multilocular lipid droplets, reduced the size of lipid droplets, and reduced the lipid content by ~43% in the adipose tissue of mice. Sesaminol also increased the expression levels of BAT-specific genes *Cidea* (~4-fold), *Elovl3* (~7-fold), and *Ucp1* (~6.5-fold). The BAT tissue of sesaminol-treated mice demonstrated ~1.5× higher rates of basal respiration compared to the untreated control animals. In high-fat diet (HFD)-fed mice, sesaminol treatment decreased weight gain by ~6% as compared to HFD control mice and enhanced glucose clearance and insulin sensitivity [148].

### 4.2. PRDM16

L-Theanine is a nonproteinogenic amino acid, a component of green tea (*Camelia sinensis*) extract, able to promote the browning of WAT by enhancing adaptive thermogenesis and increasing the expression of thermogenic genes such as *Prdm16* and *Ucp1*. Treatment of the C3H10T1/2 cell line with L-theanine in vitro induced upregulation of *Prdm16*, *PGC1a*, and *Ucp1* in a dose-dependent manner. This process is mediated through the AMPK/α-Ketoglutarate/PRDM16 axis.

In vivo, L-theanine increased the oxygen consumption rate and, consequently, enhanced mitochondrial function in WAT compared to mice in the control group. L-theanine induced the expression of the brown fat-specific genes in inguinal WAT (iWAT), epidydimal WAT (eWAT), and BAT and increased the protein levels of Prdm16, Ucp1, and PGC1α, according to immunoblotting results. In HFD-fed obese mice, L-theanine induced about a two-fold reduction in iWAT and eWAT tissue weight and, consequently, reduced body weight gain. L-theanine-treated mice also exhibited improved glucose tolerance and insulin sensitivity [149]. It is worth noting that PRDM16 knockout abolished L-theanine-induced BAT-specific gene overexpression in vitro and resulted in impaired cold tolerance both in L-theanine-treated and control mice in vivo [149]. Administration of L-theanine also increases energy expenditure, improves glucose tolerance, and enhances insulin sensitivity in mice [149].

Bexarotene (Bex) is a specific agonist of retinoid X receptors (RXR), which act downstream of PPARγ in adipogenesis. In vitro, Bex induced expression of brown adipocyte-specific genes, including *Pparg*, *Prdm16*, *Ppargc1a*, and *Ucp1*, and promoted brown adipogenic differentiation in C2C12 cells. At the same time, HX531 (an antagonist of RXR) inhibited both basal and Bex-induced brown adipogenic differentiation in C2C12 cells. In vivo, Bex at 50 mg/kg/day was orally administered to mice for 4 weeks along with a high-fat diet. Bex upregulated *Ucp1*, *Ppargc1a*, *Prdm16*, *Ppara*, *Pparg*, and *Ppard* in the adipose tissue of mice. Bex treatment reduced body weight gain in comparison with HFD-fed control mice despite similar food consumption, suggesting that Bex likely increases energy expenditure; moreover, Bex increased heat production and improved glucose sensitivity and insulin resistance, and enhanced cold tolerance [140].

Dietary long-chain omega-3 polyunsaturated fatty acids (PUFAs), in particular eicosapentaenoic acid (EPA), which is the main compound of fish oil, have an anti-inflammatory bioactive effect and potentially induce browning of adipose tissue [150]. It was shown that EPA upregulates browning markers such as *PGC1α* and *PRDM16* in a UCP1-independent manner [151,152,153]. Administration of EPA to WT and UCP-1 knockout mice resulted in improvements in insulin resistance and inflammation; however, EPA did not affect body weight or adiposity [151].

### 4.3. SIRT1/AMPK/PGC1α Axis

Curcumin is a natural curcuminoid of turmeric (*Curcuma longa*), which is safe and tolerable even at high doses (12 g/day) in humans [154]. Curcumin displays beneficial health effects, prevents weight gain, and is related to obesity and inflammation in animal models [155]. In vitro, curcumin significantly increased the expression of brown fat markers (PGC-1α, PPARγ, and UCP1) in 3T3-L1 cells and primary white adipocytes in a dose-dependent manner. Curcumin treatment enhanced mitochondrial biogenesis: the density of mitochondria and, moreover, mRNA and protein levels of PGC-1α, a key player in mitochondrial biogenesis, were markedly elevated. Curcumin treatment increased both total AMPK and phosphorylated AMPK levels; therefore, the authors hypothesized that curcumin induces browning via the AMPK-mediated pathway. This hypothesis was proved in an experiment with the treatment of adipocytes with AICAR (an activator of AMPK) and dorsomorphin (an inhibitor of AMPK). Dorsomorphin treatment abolished overexpression of *UCP1*, *PRDM16*, and *PGC-1α*, while the activator AICAR treatment resulted in elevated expression of these brown marker proteins [156]. In vivo, curcumin induced the expression of a number of brown fat-specific genes in iWAT, including *Ucp1*, *Ppargc1a*, *Prdm16*, *Dio2*, *Ppara*, and *Cidea*, and increased mitochondrial biogenesis as determined by mtDNA copy number. Curcumin reduced weight gain in C57BL/6 mice but did not affect food intake, i.e., increased energy expenditure. Curcumin-treated mice exhibited increased cold tolerance compared with control mice [154,157].

Medicarpin is a natural pterocarpan found in *Swartzia madagascariensis* and *Medicago truncatula* [158], which demonstrates different biological effects, including stimulation of bone regeneration, inhibition of osteoclastogenesis, and induction of apoptosis [159]. In vitro medicarpin treatment increased brown- and beige-fat marker expression in C3H10T1/2 cells, including *Ucp1* (2.6-fold), *Ppargc1a* (4.5-fold), *Prdm16* (2-fold), *Ppara* (2.3-fold), *Cidea* (1.9-fold), and *Elovl3* (4.8-fold). Medicarpin significantly increased the expression of certain mitochondrial genes (*Cox7a*, *Cox8b*, *Tfam*, and key mitochondrial biogenesis marker *Sirt1* (four-fold)) and increased mitochondrial mass compared to rosiglitazone. Medicarpin treatment induced AMPKα activation in a dose-dependent manner. To confirm the mechanism of medicarpin action via AMPK, a specific AMPK inhibitor, dorsomorphin, was used that abrogated medicarpin-mediated upregulation of *Pparc*, *Prdm16*, *Ppargc1a*, and *Ucp1* [160].

Palmitoylethanolamide (PEA) is a natural endocannabinoid-like lipid mediator, an amide of palmitic acid. PEA has been shown to promote the conversion of white adipose tissue to beige. PEA is able to restore sensitivity to leptin and tissue hormones [161]. The activity of PEA evaluated in 3T3-L1 cells showed increased expression of such genes specific for thermogenic adipocytes as *Ucp1*, *Ppargc1a*, *Prdm16*, and *Cox8b*. Leptin and adiponectin levels increased after PEA treatment, along with decreased secretion of the proinflammatory cytokines IL-6 and TNF-α [161].

Caffeine, often consumed in combination with other related compounds such as catechins, theobromine, and quercetin, promotes the browning of white adipocytes by upregulating the expression of brown adipocyte-specific genes and inducing lipolysis. Treatment of differentiated 3T3-L1 cells with caffeine and catechins results in suppression of lipid accumulation coupled by enhanced expression of genes coding for PPARγ, GLUT4, HSL, UCP1, and TMEM26 [162]. In addition, caffeine was shown to inhibit 3T3-L1 differentiation by disrupting mitotic clonal expansion in 3T3-L1 preadipocytes and inhibiting AKT/GSK3β signaling in differentiating 3T3-L1 preadipocytes [163]. Caffeine was able to promote *Ucp1* gene expression and enhance mitochondrial biogenesis in mouse mesenchymal stem cells [164]. In vivo studies demonstrate that caffeine consumption upregulates the expression of genes coding for UCP1, UCP2, and UCP3 in BAT and UCP2 and UCP3 in skeletal muscle, thereby promoting thermogenesis in obese yellow KK mice [165]. Ingestion of 4.5 mg·kg^−1^ of caffeine raises 3 hr postexercise energy expenditure by up to 15% [166]. Theobrownin (TB) is a main active ingredient found in pu-erh tea (*Camelia sinensis*), which stimulates lipid metabolism and, as such, contributes to body weight reduction [167]. It was shown that TB regulates lipolysis in rats and prevents the increase in serum cholesterol levels in rats on a high-fat diet [168].

Theobromine, a methylxanthine derived from cocoa beans, is known for its various health-promoting properties. Theobromine suppresses adipocyte differentiation by causing the degradation of the C/EBPβ protein through the ubiquitin-proteasome pathway. This process is mediated by the interaction with adenosine receptor A1 (AR1) and increased sumoylation of C/EBPβ [169]. Moreover, theobromine inhibits lipid accumulation and the expression of *PPARγ*, *C/EBPα*, *aP2*, and *leptin* in 3T3-L1 cells. Disruption of 3T3-L1 differentiation by theobromine is carried out through the AMPK and ERK/JNK signaling pathways [170]. In vivo administration of theobromine in HFD-fed mice leads to increased expression of key browning markers such as PRDM16 and UCP1, leading to the browning of iWAT and activating BAT [171]. Theobromine upregulates the expression of UCP1 in a PPARγ ligand-dependent manner [172].

Coffee, tea, and cocoa-derived bioactive components possessing anti-obesity effects include chlorogenic acid, trigonelline, kahweol, catechins, epigallocatechin gallate, theaflavins, thearubigins, and quercetin [173].

Quercetin, a bioactive compound present in the extract of onion peel (*Allium cepa*), contributes to adipose tissue browning via the SIRT1/AMPK signaling pathway [174,175]. Treatment of 3T3-L1 cells with quercetin derivatives in concentrations up to 25 µg/mL induced cell browning [176]. Administration of onion peel extract or 0.1% (*w*/*w*) quercetin along with HFD to C57B1/6J mice induced upregulation of brown fat markers, such as PRDM16, UCP1, Cidea, and PGC-1α in WAT. Quercetin treatment also resulted in a reduction in plasma triglyceride levels; however, it did not affect body composition or energy expenditure in mice [176,177].

Phytol, the most abundant acyclic isoprenoid, used as a precursor for synthetic vitamin E production, was able to decrease the body weight gain in mice through stimulation of inguinal WAT browning [178]. At the same time, phytol administration led to an increase in the expression of genes characteristic for brown adipocyte (*UCP1*, *PRDM16*, *PGC1α*). Thorough in vitro studies demonstrated that a 100 μM phytol solution increased mitochondria content and oxygen consumption in the differentiated 3T3-L1. It also stimulated brown adipogenic differentiation and the formation of brown-like adipocytes by promoting mRNA and/or protein expression of brown adipocyte markers (UCP1, PRDM16, PGC1α, Cidea, and Elovl3) and beige adipocyte markers (CD137 and TMEM26). Meanwhile, inhibition of AMPKα with Compound C abolished phytol-stimulated brown adipogenic differentiation and the formation of brown-like adipocytes, confirming that phytol is acting through the AMPKα signaling pathway in 3T3-L1.

Previously discovered activities of small molecules, in particular micronutrients and phytochemicals, on browning of adipose tissue confirm their contribution to the formation and functioning of beige adipose tissue in adults. It is worth noting that the molecular mechanisms described above explain many but not all observed effects.

## 5. Disruption of White-to-Beige Transdifferentiation

There is a group of chemical compounds that have been shown to disrupt the normal functioning of endocrine regulation. Their specific ability to distort the regulatory function is linked to their hormone-like structure [179]. Some endocrine-disrupting chemicals are known to derail adipogenesis and inhibit the browning of adipose tissue [180]. Most of the discussed compounds are environmental contaminants and industrial pollutants described as endocrine disruptors for their ability to impair processes of systemic regulation [181]. More than twenty years ago, Jerrold Heindel reasonably hypothesized the influence of endocrine-disrupting chemicals (EDCs) on obesity, as nearly every aspect of metabolism is regulated by the endocrine system [182]. Endocrine disruptors act explicitly or implicitly as obesogens by defecting adipogenesis and fostering lipid accumulation [183].

EDCs interact with nuclear hormone receptors important for the development of white adipocytes. One of the first well-studied endocrine disruptors was the drug diethylstilbestrol, a synthetic estrogen widely prescribed from the 1940s through to the 1970s. It was also the first chemical to confirm the proposed theory of obesogens in vivo, as neonatal exposure caused an increase in body fat [184]. These effects of pharmaceutical estrogens selectively binding the estrogen receptors are mediated through downstream-regulated genes and are predominantly sex-dependent [185]. Activation of ERα led to murine adipose-derived stromal cell differentiation towards the white adipocyte lineage; instead, ERα deficiency determined the cell fate towards smooth muscle or brown adipocytes [186]. The latter is possibly the mechanism underlying the sex-dependent manner of adiposity and, specifically, adipocyte proliferation differences.

Compounds widely used for plastic production, such as bisphenol A (BPA), were shown to alter the adipose tissue metabolism as well [187]. BPA is found in plastic bottles and food containers, as it is used for the production of polycarbonate plastics and epoxy resins [188]. It is one of the main obesogens, which means it can disrupt normal metabolic processes and contribute to obesity.

Studies have shown that exposure to BPA can induce preadipocyte differentiation, therefore promoting adipocyte hypertrophy and leading to increased fat accumulation [189]. Exposure to BPA disrupts normal adipocyte development by inducing differentiation through a non-classical estrogen receptor pathway rather than through glucocorticoid stimulation. This leads to an increase in adipose tissue mass in vivo and hypertrophic adipocytes in males, which results in increased body weight [190]. Moreover, BPA has been linked to the induction of proinflammatory pathways and upregulation of the expression of cytokine genes such as IL1β, IL6, and TNFα. Thus, BPA can exert effects associated with a chronic low-grade inflammatory state of adipose tissue [191].

The reported xenoestrogenic effect of BPA largely impairs development and leads to adverse effects in reproduction [192]. The mechanism of endocrine disruption exerted by BPA in most conditions results in transgenerational effects [193]. Recently, BPA was considered particularly dangerous by the Committee of the Member States of the European Chemicals Agency because of its reproductive toxicity and its endocrine-disrupting properties for human health and the environment. Since then, it has been substituted by other compounds from the bisphenol family, such as BPS, BPF, and BPAF [194].

New BPA-replacement compounds may be even more harmful than the original BPA. For example, most highly prevalent “BPA-free” plastics contain bisphenol S (BPS). It has been reported that the presence of BPAF and BPS is associated with such metabolic disorders as gestational diabetes [195]. The evidence is clear that the xenoestrogenic activity of bisphenols leads to suppression of beige adipocyte formation, which is important for thermogenesis and energy expenditure [196,197]. By inhibiting the formation of beige adipocytes (Figure 2), bisphenols disrupt the important metabolic function of beige adipose, contributing to weight gain and systemic metabolism.

Peroxisome proliferator-activated receptors are hormone receptors, described earlier as molecular targets for synthetic thiazolidinediones. Some EDCs were demonstrated to have effects on PPAR activity. Organotin compounds used in antifouling paints and as stabilizers in plastics can disrupt adipocyte differentiation and function. In particular, organic derivatives of tin (IV), tributyltin (TBT), and triphenyltin (TPT), having a completely different structure from that of 9-cis retinoic acid (9cisRA), the endogenous ligand of the Retinoid X Receptor, mimic its action and induce heterodimerization of PPARγ:RXR complexes [198,199]. Organotins have been shown to induce PPAR activity showing the absence of specific transcriptional marks characteristic for thermogenesis [200,201]—they inhibit the browning of white adipose tissue [202].

Another group of pollutants that pose a threat to adipose tissue health are phthalates. Phthalates are a group of chemicals used in plastics, personal care products, and food packaging. Bis(2-ethylhexyl)phthalate (DEHP) is the plasticizer most widely used to manufacture various soft poly(vinylchloride) plastics, especially tubes for medical equipment, while mono(2-ethylhexyl)-phthalate (MEHP) is its metabolite [203]. Both MEHP and DEHP were shown to disturb adipogenesis in cell culture models by hyper-activating PPARγ [204,205]. Phthalates can activate PPAR:RXR heterodimerization [206]. Moreover, it was demonstrated that the synergy of both MEHP and 9-*cis*-RA and a combination of suboptimal concentrations of MEHP and a natural ligand of PPAR (15d-PGJ2) resulted in activation of the PPARγ:RXR dimer towards PPRE-DNA binding greater than that of either compound alone [207]. Structural studies show that the MEHP molecule binds the activating function-2 (AF-2) sub-pocket and the hydrophobic ligand-binding pocket [208], resembling interactions between PPARγ and endogenous fatty acids binding. Thus, the contribution of phthalates to activation of the nuclear receptor PPARγ is undeniable and often leads to disruption of regulation. Apart from adipose tissue, PPARs are expressed in the central nervous system and in organs of the reproductive system, i.e., the gonads (testis and ovary), uterus, prostate, mammary gland, and pituitary gland [209]. Even so, some researchers still raise the question of whether phthalates have a beneficial or malicious effect on thermogenic adipose tissues [210]. It turns out that there is a need for careful interpretation of ambiguous moments, as it was reported that there was an increase in browning marker gene expression (*Pparg*, *Ppargc1a*, and *Ucp1*) in WAT and higher amounts of BAT [211], but at the same time, increased body weight gain in HFD-fed mice [212]. Therefore, disrupting the normal activity of PPAR phthalates can cause excess adiposity in childhood and later in life [213,214]. Developmental exposure to phthalates has been shown to decrease UCP1 protein expression and BAT activity, inducing significant hypothermia and leading to hyperphagia in male mice [215]. This observation confirms that EDCs such as phthalates inhibit the beigeing of white adipose tissue.

Compounds that counteract the beigeing process include polychlorinated biphenyls and dioxins. Polychlorinated biphenyls (PCBs) are a group of industrial chemicals that were banned in the 1970s but are still present in the environment due to their persistence. Exposure to PCB126 was shown to impair adipogenesis and alter adipocyte metabolism [216]. PCB126-induced disruption resulted in a significant reduction in fully differentiated adipocytes due to PPARγ inhibition. The reduction in PPARγ transcript levels observed was accompanied by the activation of AhR by PCB126. PCBs have a dioxin-like structure and therefore bind to and activate the aryl hydrocarbon receptor (AhR). Moreover, when the cells were exposed to PCB126 during differentiation, the browning of white adipose tissue was inhibited by disrupting mitochondrial uncoupling and energy expenditure [217]. Differentiated adipocytes exposed to PCB126 had a reduced ability for UCP1-related uncoupling.

Dioxins are far beyond other xenobiotics in their toxic effects, specifically those related to metabolic disorders like obesity, diabetes, and metabolic syndrome [218]. This is aggravated by the fact that dioxins accumulate in the adipose tissue of mammals due to their high lipophilicity. 2,3,7,8-Tetrachlorodibenzo-*p*-dioxin (TCDD) is one of the most potent compounds of the dioxin class, and the spectra of its toxicity include metabolic disorders emerging through direct action on adipocytes or the induction of local inflammation of the adipose tissue [219].

Brown adipose tissue was identified as a target tissue for TCDD almost 40 years ago, and the direct effects of dioxins were studied in rats [220]. Thereafter, the experiments with TCDD were limited due to its high toxicity, but a model was established to study the effects of dioxins’ release from adipose. The epididymal fat pads of dioxin-exposed mice were collected and grafted on the back skin of untreated recipient animals [221]. Redistribution of TCDD to other tissues led to massive changes in gene expression and was found to be completely dependent on AhR activation.

### AhR

The aryl hydrocarbon receptor belongs to the Per-ARNT-Sim (PAS) family of transcription factors, known as sensors of environmental signals [222]. AhR is a transcription factor that was originally identified as a sensor of dioxins [223].

AhR resides in the cytoplasm when not activated in a majority of cell types forming the complex with HSP90 (Heat Shock Protein 90), co-chaperone p23 and its partner, the aryl hydrocarbon receptor-interacting protein (AIP also known as ARA9) [224,225]. The AIP protein, structurally related to the FK506-binding protein class of immunophilins, acts as a chaperone, presumably maintaining properly folded AhR in the cytosol and improving the stability, subcellular localization, recognition of ligand and, subsequently, efficient translocation.

When AhR binds a ligand, it translocates into the nucleus, where, in the form of a heterodimer with AhR nuclear translocator (ARNT) induces the expression of genes involved in various biological responses. Specifically, the AhR:ARNT heterodimer binds enhancers known as dioxin-responsive elements (DREs or XREs) in order to induce the expression of genes encoding xenobiotic-metabolizing enzymes [226]. This pathway represents an adaptive response that allows for the detoxification of a wide variety of compounds with polycyclic aromatic structures by xenobiotic-metabolizing enzymes. Therefore, the number of AhR-induced genes includes *Cyp1a1*, *Cyp1a2*, *Cyp1b1*, and a negative regulator of AhR signaling known as the AhR repressor (AhRR), which competes with ARNT in the process of heterodimerization and represses the activation of AhR-dependent genes [227].

TCDD binds to the AhR, inducing the transcription of xenobiotic-metabolizing enzymes as well. Despite the induction of these enzymes, the TCDD and other persistent organic pollutants are metabolized insufficiently, and their elimination or degradation is slow; therefore, the accumulation in adipose is observed [228].

*AhR* is expressed in adipocytes [229], and specifically, in the model cell line 3T3-L1. The level of AhR was shown to decrease in 3T3-L1 cells during differentiation, as well as its binding activity towards the response elements and the response to TCDD. AhR has earned increased attention for its possible involvement in the regulation of body weight, adipose tissue expansion, and lipid homeostasis in vivo [230,231]. Although some data showed that activation of AhR led to lipogenesis suppression, mouse embryonic fibroblasts isolated from AhR-deficient mice displayed enhanced synthesis of triacylglycerols [232]. TCDD-induced activation of AhR was able to inhibit adipogenesis by suppressing PPARγ activity and impairing the adipogenesis of 3T3-L1 in vitro. The absence of such an effect in mouse embryonic fibroblasts isolated from AhR−/− mice highlighted the role of AhR in hormone-induced adipogenesis, suggesting its role as an early regulator of adipocyte differentiation.

Recent in vitro experiments demonstrated that AhR overexpression suppressed adipocyte differentiation through reduced PPARγ stability and, on the contrary, siRNA-mediated Ahr gene silencing led to adipocyte differentiation in 3T3-L1 cells [233]. Furthermore, it was found that AhR functions as the substrate receptor in CUL4B-RING E3 ubiquitin ligase targeting PPARγ ubiquitination by binding two lysine sites on residues 268 and 293.

Animal research unexpectedly revealed an increased body weight in mice with adipocyte-specific AhR deficiency compared to wild type mice. The phenotype demonstrated an increased fat mass and adipose tissue inflammation, accompanied by a decreased glucose tolerance when fed a high-fat diet [234]. Instead, the whole-body deficiency of AhR protected mice from high-fat diet-induced obesity through increased energy expenditure [230]. Moreover, AhR deficiency did not alter insulin sensitivity in adipose or muscle tissues. The expression of the major thermogenic gene, uncoupling protein 1 (*Ucp1*), in brown adipose tissue and genes responsible for mitochondrial β-oxidation in muscle were significantly higher in AhR (−/−) and AhR (+/−) mice compared with wild type mice. Another study recently revealed a sex-dependent phenotype of AhR depletion specific for mature adipose tissue (CadKO) cells [235]. CadKO females had a lean phenotype and healthy adipose–hypothalamic crosstalk. Interestingly, HFD-induced leptin rise was reduced in CadKO females, while the leptin receptor was increased in the energy regulatory regions of the hypothalamus, suggesting an increase in leptin sensitivity. The expression level of estrogen receptor α (ERα) was increased in CadKO female adipose tissue and the hypothalamus. CadKO males displayed a delayed progression of obesity and insulin resistance. In males, beneficial effects of adipose-specific AhR depletion were mediated through the maintenance of healthy crosstalk between adipocytes and immune cells: proinflammatory adipocytokines’ (such as TNFα, IL1β, IL6) secretion was improved and inflammatory macrophage infiltration into adipose was reduced. Overall, adipose-specific knockout of AhR gene improved weight control and systemic glucose homeostasis in a high-fat-diet-induced condition with more pronounced effects in females.

We suppose that AhR can regulate the browning of adipose through tissue-resident immune cells. This suggestion was confirmed by experimental data showing that AhR plays an important role in regulating ILCs. *AhR* is expressed in ILC2s at the highest levels, and its pharmacological activation could suppress the function of this distinct population of lymphoid cells [236]. Remembering that ILC2s regulate adipose function and metabolic homeostasis through the induction of beigeing [50] it is highly likely that AhR depletion in these cells may prevent obesity.

Several phytochemical and dietary compounds are able to modulate physiological processes by antagonizing AhR signaling [237]. In particular, isoprenoids and phenylpropanoids [238], quercetin, kaempferol [239], curcuminoids, and coumarins [240], were demonstrated to antagonize aryl hydrocarbon receptor. A number of AhR ligands, such as curcumin [241,242], resveratrol [243,244,245], and quercetin [246], repeatedly demonstrated inhibition of TCDD-induced gene expression. A specific group of monoterpenoids, carvones, the constituents of essential oils of dill, caraway, and spearmint, was reported to be noncompetitive, insurmountable antagonists of AhR [247]. Binding of such natural ligands inhibits the heterodimerization of AhR with ARNT and does not lead to *Cyp1a1* gene induction [247]. In addition, synthetic small molecules were developed that act as powerful AhR antagonists: CH-223191 [248], 6,2,4-trimethoxyflavone [249], and GNF351 [250], including two molecules being tested in clinical trials BAY2416964 [251] and IK-175 [252].

This section is an example that one can be convinced that something “bad”, exhibiting direct toxic activity, can be turned into “good”—a tool for studying the molecular mechanisms of the regulation of inflammation in adipose tissue. The critical role of AhR in PPARγ stability represents the potential of AhR as a therapeutic target for metabolic disease treatment. A multitude of AhR natural antagonists have been described, encouraging the development of new small molecule compounds exerting antagonism of AhR. We think that the discussed compounds and molecular mechanisms can possibly give a clearer insight into the nature of the problem.

## 6. Methods of White-to-Beige Conversion

White-to-beige conversion occurs naturally, but also can be induced artificially in many ways and approaches. As was already noted, the most common methods are based on the application of small molecules that initialize tissue reprogramming. Bexarotene and rosiglitazone are the most used and well-known agonists of PPARγ and its partner retinoid X receptor that regulate the transformation of C2C12 cells to beige fat tissue [140]. Discovering new agonists of this receptor can be a perspective for future research. In addition to RXR agonists, several small molecules such as rosiglitazone, BMP7, and forskolin can be used as a part of a “chemical cocktail” that allows the enforcing of the overexpression of transcriptional factors such as C/EBP-β, C-MYC [253].

In a similar way, the “gene cocktail” PRDM16, BMP7, and PGC1A can be applied for transfection with ultrasound-targeted microbubble destruction methods [254]. In this method, lipid microbubbles are filled with a plasmid mixture burst under ultrasonication of target tissue and gas carries DNA to cells. The potency effect of such gene delivery was measured with RT-PCR by the overexpression of *UCP1* gene after 24 h. The authors note that the level of UCP1 expression with three genes is higher than with PRDM16 alone, but a single gene can also be used for gene therapy applications.

**Figure 3:**
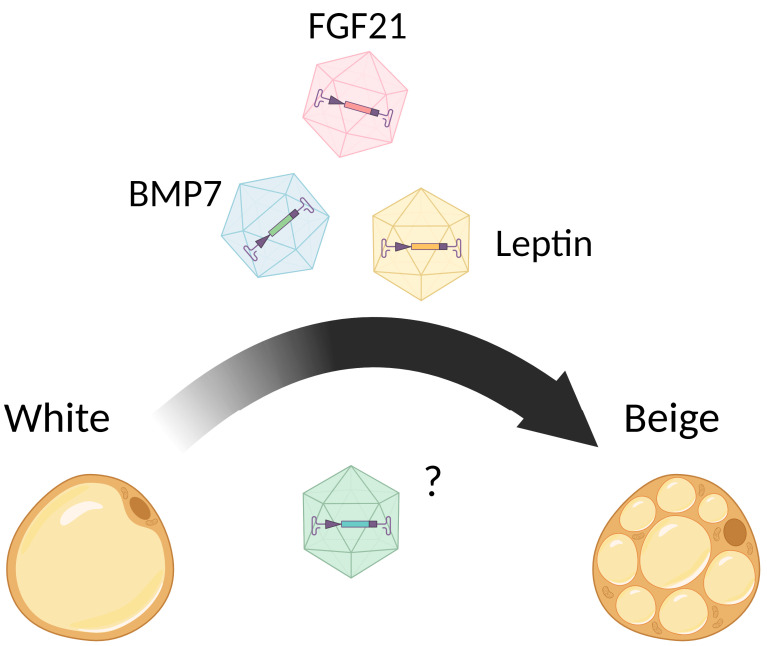
AAV vectors in white-to-beige conversion.

Viral gene delivery is the most common type of gene therapy approach used to convert WAT into beige adipose, to achieve stable ubiquitous or tissue-specific overexpression of target genes and increase energy expenditure. Different types of noninfective recombinant viruses, including adenovirus, retrovirus, lentivirus, and adeno-associated virus (AAV) were used to direct the transgene expression in adipose tissue [255]. However, it was shown, that adenovirus, retrovirus, and lentivirus are immunogenic and do not provide the long-term transgene expression [256,257,258,259]. Moreover, gamma-retroviruses and lentiviruses are characterized by an increased risk of carcinogenicity and genomic instability due to their ability to integrate into the host genome [260,261]. In contrast, AAV vectors (Figure 3) are characterized by low immunogenicity, low toxicity, and carcinogenicity risk, are present in the nucleus in the form of episomes, and provide stable and long-term transgene expression [255,262].

AAV vectors could be injected intravenously [263,264] or locally (for example, into WAT depot [265], intracranial [266]). Systemic delivery requires a higher dose than is used when the virus is injected locally [262,264,267]. Moreover, local injection or tissue-specific expression is associated with fewer adverse events. Targeting WAT provides an opportunity for surgical removal of this organ in case some severe adverse events happen [268]. Tissue-specific expression is achieved by using tissue-specific promoters (i.e., the adipose tissue-specific adiponectin promoter [264], the liver-specific human α1-antitrypsin (hAAT) promoter [262], the liver-specific albumin promoter [267]). However, the disadvantage of tissue-specific promoters is that they are weaker in terms of achieving high transgene expression than ubiquitous (hybrid cytomegalovirus enhancer/chicken-actin (CBA or CAG)) or cytomegalovirus promoters (CMV). Moreover, due to the limited capacity of AAV, mini-versions of the promoters are used (adipocyte protein 2 promoter (mini/aP2) for WAT and uncoupling protein 1 promoter (mini/UCP1) for BAT) [269]. Moreover, to de-target viral vector expression from the liver or heart in order to mitigate the risk of adverse events, target sequences for local miR-122a or miR-1 are introduced into the 3′-UTR of the AAV expression cassette [264,265]. One of the first attempts at adeno-associated virus application for adipose tissue transfection was performed in 2006 by Mizukami [268]. In this work, the delivery of the erythropoietin gene into adipose cells using different AAV serotypes (AAV1–5) was studied. As a result, the AAV1 serotype demonstrated the highest transduction efficiency, which was evaluated according to the erythropoietin expression level in the blood measured using qPCR. The removal of transduced adipose tissue resulted in normalization of the erythropoietin level in the blood; this result supported the efficiency and safety of the approach. A number of experiments with the new generation of recombinant serotypes (AAV5–9) showed the highest transduction efficiency of AAV8, which resulted in the highest level of GFP expression under the control of the adiponectin promoter in brown adipose that was visualized with immunoblotting and fluorescent microscopy [264]. When the most efficient AAV serotype was found, researchers made a successful attempt to deliver the leptin gene to the adipose tissue of ob/ob mice as an approach to correct congenital generalized lipodystrophy, associated with severe metabolic imbalance, the virtual lack of adipose tissue, hepatic steatosis, and related cardiovascular conditions. The AAV vector encoding leptin gene under the control of adiponectin gene promoter was constructed. Moreover, to decrease non-specific transgene expression in liver, the sequence of miR-122 was introduced to the construction. I.v. injection of 1 × 10^12^ vg of the obtained vector to ob/ob leptin-deficient mice resulted in a decrease in weight gain, and an improvement in hyperinsulinemia and glucose tolerance. Therefore, the authors proved the concept that adipose-specific leptin overexpression can correct gene deficiency and related disease in a mouse model [264].

In another study, intraperitoneal injection of AAV vector, encoding the gene of leptin under the control of a potent CBA promoter (dose: 4 × 10^10^ vg), resulted in high level of transgene expression in visceral adipose tissue. AAV also contained the second expression cassette encoding miRNA targeting the woodchuck posttranscriptional regulatory element (WPRE) sequence, which was present only in the transgene expression cassette, under the control of the liver-specific albumin promoter. This miRNA enabled the restriction of off-target leptin overexpression in the liver. The injection of AAV, encoding the leptin gene, to ob/ob mice resulted in a sharp decrease in food intake, the normalization of body weight, complete rescue of the impaired glycemic control, and an increase in oxygen consumption and locomotor activity [267].

Another attempt at a gene replacement strategy using AAV8 was utilized in a mouse model with congenital lypodistrophy caused by seipin knockout [263]. This disease is associated with mutations in the BSCL2 gene (from the alternative name of congenital lypodistrophy: Berardinelli-Seip syndrome), which is coding for the protein seipin. A single i.p. injection of 1 × 10^12^ vg AAV, encoding GFP under the constitutive CMV promoter, resulted in GFP expression in WAT and BAT. The treatment of seipin knockout mice with 1 × 10^12^ vg AAV8, encoding human BSCL2 gene (AAV8-CMV-hBSCL2), resulted in an increase in body weight gain, the recovery of hyperglycemia, hepatomegaly, and insulin resistance, and the partial restoration of adipose tissue.

Fibroblast growth factor 21 (FGF21) is a peptide hormone contributing to energy homeostasis regulation. FGF21 was shown to be a promising compound for diabetes mellitus therapy [270]. AAV vectors encoding the FGF21 gene under the control of the liver-specific hAAT promoter were constructed [271]. A total of 5 × 10^10^ vg of the resulting vector or non-coding AAV vector was iv injected into ob/ob and HFD-fed mice. The treatment of HFD-fed mice with 5 × 10^10^ vg AAV8-hAAT-FGF21 resulted in progressive weight loss (the body weight at the end of the study was similar to the baseline values measured before the initiation of HFD), and improvements in HFD-associated WAT hypertrophy and inflammation, hepatic steatosis, and fibrosis. AAV8-hAAT-FGF21 treatment induced BAT activation, which was demonstrated by a dose-dependent increase in UCP1 level. However, the authors did not observe the appearance of multilocular adipocytes (i.e., browning) in WAT. Treated with 5 × 10^11^ vg AAV8-hAAT-FGF21, ob/ob mice also demonstrated decreased WAT inflammation, an improvement in hepatic steatosis, and a marked reduction in the total liver triglyceride and cholesterol content.

A few successful attempts at BMP7 overexpression using viral vectors were made [259,262,265]. Bone morphogenic protein 7 (BMP7) belongs to the superfamily of transforming growth factors β, which contribute to such vital processes as cell proliferation, differentiation, and apoptosis [272]. BMP7 was shown to promote brown adipocyte differentiation from multipotent mesenchymal stem cells [259]. The first attempts of BMP7 overexpression were made using adenovirus (Ad) vectors [259]; Ad vector transduction resulted in an increase in brown adipose tissue mass, increase in energy expenditure, and weight gain reduction. However, due to the high immunogenicity of Ad vectors, long-term transgene overexpression could not be reached.

A number of works on the overexpression of BMP7 were conducted in the scientific group of Prof. Fatima Bosch [262,265]. First, AAV8 vectors encoding BMP7 gene under the control of the ubiquitous CAG promoter and under the control of the liver-specific hAAT promoter were created [265]. To de-target transgene expression from the liver and heart, target sequences for miR-122a and miR-1 were introduced into the 3′-UTR of AAV vector expression cassettes. Injection of the obtained vectors (1 × 10^12^ vg) into the epidymal WAT of ob/ob mice reduced WAT inflammation, and improved hepatic steatosis and insulin resistance, but did not induce brown adipogenesis, as was shown by the unchanged level of expression of *Ucp1* and *Ppargc1a* in iBAT and iWAT.

In another work [262], an AAV8 vector encoding the BMP7 gene under the control of a synthetic hybrid liver-specific promoter composed of the hepatocyte control region enhancer from the apolipoprotein E gene (ApoE) and the hAAT promoter was constructed. Intravenous injection of 1 × 10^12^ vg AAV-BMP7 vector into HFD-fed obese mice resulted in the normalization of body weight and liver weight within a few weeks. Upregulation of thermogenic markers *Ucp1*, *Cidea*, and *Ppargc1a* was demonstrated in iWAT of AAV-BMP7-treated animals, which indicated the activation of non-shivering thermogenesis. Moreover, hepatic steatosis and insulin resistance were mitigated in AAV-BMP7-treated mice. The induction of non-shivering thermogenesis and improvement in insulin sensitivity were also demonstrated in ob/ob mice after treatment with AAV-BMP7.

Anderson et al. [266] performed a study on the overexpression of the hypotalamic protein TrkB.FL, related to metabolic homeostasis and autism spectrum disorder (ASD) development, in the hypothalamus of BTBRT+Itpr3tf/J mice (on a normal chow diet and HFD-fed), which serve as the model of ASD. In this study, an AAV2 vector containing CMV enhancer, chicken β-actin (CBA) promoter, and the TrkB.FL gene was injected intracranially (into the hypothalamus) at a dose of 2.5 × 10^9^ vg/side to obtain a high level of constitutive TrkB.FL overexpression. As a result, transgenic mice both on NCD and HFD demonstrated a decreased percent body weight gain compared to normal BTBRT+Itpr3tf/J mice, despite the fact that they consumed significantly more food. AAV2-TrkB.FL-transfected NCD mice also showed improved glucose tolerance.

Despite that fact, the majority of studies were focused on the overexpression of brown adipose tissue activators, and in the last few years, a number of experiments have been performed where gene knockout resulted in an increase in energy expenditure and an improvement in metabolic outcomes. A number of reports were published demonstrating that gene knockout results in the activation of the browning program and an increase in energy expenditure. For example, knockout of NAD^+^-dependent deacylase sirtuin 7 (SIRT7) resulted in the upregulation of UCP1, and an increase in body temperature and energy expenditure in mice [273]. Selective knockout of Rbm43 in mouse adipocytes increased PGC1⍺ translation, mitochondrial biogenesis, and adipose thermogenesis [274]. Genetic or pharmacological inactivation of key upstream kinases of the Hippo signaling pathway, STK3 and STK4, increased the expression level of UCP1 in BAT and WAT, increased mitochondrial mass and conferred resistance to metabolic dysfunction induced by HFD, and increased mitochondrial content in adipose tissue and mitochondrial oxidative respiration [275]. Ablation of Sam68 (Src-associated-in-mitosis-of-68 kDa) in adult mice led to body weight reduction [276]. Adipose thermogenesis and energy expenditure were also increased after knockout of the Krüppel-associated box (KRAB) domain-containing zinc finger protein ZFP961, which serves as a potent repressor of the thermogenic program [277].

From the presented data, we anticipate the use of AAVs as one of the safest known vectors for gene therapy [278]. Most of the experimental studies show the effectiveness of AAV usage (Table 1), but regarding the limitations of the study, these findings should be viewed with caution. It is known that even adeno-associated viral vectors have unintended side effects [279]. In order to weaken these adverse events and improve the scientific significance of the experiments, we expect the utilization of different optogenetic switches developed earlier [280]. Currently, we are developing AAVs coding for transcription factors able to stimulate white-to-beige conversion both in vitro and in vivo.

## 7. Conclusions

Obesity, one of the major problems of modern medicine, has a multifactorial pathogenesis. At the moment, a large number of small molecules have been evaluated for their ability to trigger different mechanisms of adipocyte conversion through indirect activation of signaling and transcriptional cascades, including the activation of pivotal regulators of transcription that control important metabolic pathways. In order to identify possible factors influencing white-to-beige conversion occurring in adipose tissue, we examined a variety of cellular and molecular determinants. Additionally, we described the action of phytochemical and dietary molecules affecting the transdifferentiation process in connection with molecular targets, with a specific focus on transcription factors. We spotted that the influence of pollutants and environmental disruptors on adipose tissue, which is relevant in the modern postindustrial milieu, is based on their activity towards transcription factors. Additionally, we noticed that some of the reported natural ligands antagonizing AhR were previously discussed in the “Small Molecule Compounds” section as known white-to-beige inducers. It is possible that the phenomenon of these natural phytochemicals and dietary molecules is based on their ability to suppress the transcriptional activity of AhR:ARNT and, in consequence, stabilize PPARγ. Specifically, we highlighted the possible use of AhR as a therapeutic target for metabolic disease treatment.

Existing state-of-the-art gene therapy offers several opportunities to stimulate the conversion of WAT into beige adipose tissue. As we noticed, most of the anti-obesity gene therapy approaches exploit the possibility of inducing white-to-beige adipose transition by overexpressing the genes coding for secreted factors, such as leptin, FGF21, or BMP7. However, gene therapy drugs based on the induction of single transcription factors may have fundamentally different features. Firstly, the use of AAV coding for transcription factors expands the scope of the drug effects, and since the transcription factors stimulating white-to-beige conversion are well-known, it makes it possible to exert a high-precision impact on cells. Secondly, the use of nucleic acid-based drugs changes the functioning of the cell at the level of mRNA transcripts, which are regulated by intracellular mechanisms, and has fewer off-target effects compared to the use of secreted proteins and small molecules.

Another protective mechanism that controls the process of adipogenesis at the systemic level is endocrine regulation, which is much more susceptible to influence from xenobiotic substances. In this case, adipogenesis is influenced not only by small-molecule-based drugs but also by other substances from the external environment. The influence of bisphenols, phthalates, and, widely used in modern industry and agriculture, dioxin-like compounds, poses a great threat to health. In addition to the generally recognized toxic effects known for each of these substances, we think that their intake with food, air, and water results in further problems. The accumulation of these substances in adipose, leading to prolonged adipogenesis impairment, can also affect other processes in the organism, resulting in chronic intoxication. Nevertheless, both adipocytes and adipose tissue-resident immune cells express AhR, which is responsible for dioxin sensing. Targeted ablation of this transcription factor can normalize the process of adipogenesis and suppress the proinflammatory activity of resident immune cells. Thus, gene therapy drugs that target transcription processes open up broad prospects for obesity treatment and metabolic improvement, including the reduction in the harmful effects of environmental factors.

Hereby, we summarize the current knowledge about white-to-beige adipose conversion, concluding that both small molecule medicinal chemistry and gene therapy are perspectives for the development of new strategies to overcome insulin resistance and counteract obesity.

## Figures and Tables

**Table 1:** Gene therapy and genetic models (KO) targeting adipose tissue.

	Target Gene	AAV Serotype (Genetic Therapy Tool)	Expression Cassette	Dose	Administration Route	Outcome	Reference
Overexpression	Erythropoietin	AAV1–5	CMV-Epo	6 × 10^11^ vg	s.c.	Efficient delivery of the erythropoietin gene into adipose tissue resulted in an increase in blood Epo level.	[268]
	*Leptin*	AAV2/8	Adipo-Lep-miR122 (8x)	1 × 10^12^ vg	i.v.	Successful delivery of the leptin gene to AT of ob/ob mice, decrease in weight gain, an improvement in hyperinsulinemia, and glucose tolerance.	[264]
	*Leptin*	AAVRec2	Albumin promoter-Lep-miR-WPRE-CBA-leptin-WRPE	4 × 10^10^ vg	i.p.	Decrease in food intake, normalization of body weight, normalization of glycemic control, increase in oxygen consumption, and locomotor activity in ob/ob mice.	[267]
	*BSCL2*	AAV8	CMV-hBSCL2	1 × 10^12^ vg	i.v.	Normalization of hyperglycemia and severe insulin resistance in seipin-deficient mice.	[263]
	*FGF21*	AAV8	hAAT-FGF21	5 × 10^10^–5 × 10^11^ vg	i.v.	Weight loss, improvement of WAT inflammation, hepatic steatosis, and fibrosis in HFD-fed mice; improvement of WAT inflammation, hepatic steatosis, and reduction in the total liver triglyceride and cholesterol content in ob/ob mice.	[271]
	*BMP7*	AAV8	CAG-BMP7; hAAT-BMP7	1 × 10^12^ vg	Intra eWAT injection	Efficient de-targeting of the transgene from liver and heart; improvement of hepatic steatosis and insulin sensitivity in ob/ob mice. BMP7 overexpression in WAT did not induce brown adipogenesis.	[265]
	*BMP7*	AAV8	Hybrid hAAT-BMP7	1 × 10^12^ vg	i.v.	Upregulation of brown fat markers, induction of non-shivering thermogenesis, normalization of body weight, improvement of hepatic steatosis and insulin resistance in HFD-fed and ob/ob mice.	[265]
	*TrkB.FL*	AAV2	CBA-TrkB.FL	2.5 × 10^9^ vg/ side of hypothalamus	i.c.	Decrease in percent body weight gain and improvement of glucose tolerance in BTBRT+Itpr3tf/J mice in NCD and HFD.	[266]
Knockout	*SIRT7*					Upregulation of UCP1, increase in body temperature, and energy expenditure.	[273]
	*STK3* and *STK4*					Increased *UCP1* expression level in BAT and WAT, increase in mitochondrial mass, and mitochondrial oxidative respiration in adipose tissue.	[275]
	*Rbm43*					Upregulation of PGC1⍺, increase in mitochondrial biogenesis and adipose thermogenesis.	[274]
	*Sam68*					Prevention of high-fat-diet-induced weight gain and insulin resistance.	[276]
	*ZFP961*					Increase in adipose thermogenesis and energy expenditure.	[277]
